# Clinical significance in the number of involved lymph nodes in patients that underwent surgery for pathological stage III-N2 non-small cell lung cancer

**DOI:** 10.1186/1749-8090-6-144

**Published:** 2011-10-25

**Authors:** Takeshi Hanagiri, Masaru Takenaka, Soich Oka, Yoshiki Shigematsu, Yoshika Nagata, Hidehiko Shimokawa, Hidetaka Uramoto, Fumihiro Tanaka

**Affiliations:** 1 Second Department of Surgery, School of Medicine, University of Occupational and Environmental Health, Kitakyushu 807, Japan

**Keywords:** non-small cell lung cancer, surgical resection, mediastial lymph node metastasis, number of involved lymph nodes, skip metastasis, postoperative prognosis

## Abstract

**Purpose:**

This study investigated whether the number of involved lymph nodes is associated with the prognosis in patients that underwent surgery for pathological stage (p-stage) III/N2 NSCLC.

**Subjects:**

This study evaluated 121 patients with p-stage III/N2 NSCLC.

**Results:**

The histological types included 65 adenocarcinomas, 39 squamous cell carcinomas and 17 others. The average number of dissected lymph nodes was 23.8 (range: 6-55). The average number of involved lymph nodes was 5.9 (range: 1-23). The 5-year survival rate of the patients was 51.0% for single lymph node positive, 58.9% for 2 lymph nodes positive, 34.2% for 3 lymph nodes positive, and 30.0% for 4 lymph nodes positive, and 20.4% for more than 5 lymph nodes positive. The patients with either single or 2 lymph nodes positive had a significantly more favorable prognosis than the patients with more than 5 lymph nodes positive. A multivariate analysis revealed that the number of involved lymph nodes was a significant independent prognostic factor.

**Conclusion:**

Surgery appears to be preferable as a one arm of multimodality therapy in p-stage III/N2 patients with single or 2 involved lymph nodes. The optimal incorporation of surgery into the multimodality approach therefore requires further clinical investigation.

## Introduction

More than 1.6 million new cases of lung cancer are diagnosed worldwide each year, causing approximately 1.3 million deaths annually and representing the highest mortality rate in comparison to any other major malignancies [1,2]. A surgical resection remains the mainstay for patients with early stage non-small cell carcinoma (NSCLC) [3]. However, lung cancer patients are often diagnosed with advanced disease due to the aggressiveness of this type of cancer [4,5]. A careful staging workup is very important to determine the optimal treatment strategy. Chemotherapy and radiotherapy is the current standard of care for patients with locally advanced (stage IIIA and stage IIIB) NSCLC. However, regardless of the total dose of radiation and the optimal chemotherapy, the outcome of stage III patients remains poor, with a median survival of 10-15 months, and 5-year survival rates of only 5-15% [6,7].

It is necessary to establish a treatment strategy to improve their prognosis of pathological stage (p-stage) III/N2 patients. Surgical intervention still plays a crucial role in selected cases, for achieving better loco-regional control and their favorable prognosis [8]. Generally, the results of surgical treatment as a multimodality therapy for pathological stage III are not satisfactory, because the 5-year survival rates range from 20 to 30% [9,10]. The range in the survival of stage III NSCLC associated with various prognostic factors suggests that patients at the N2 stage are a heterogeneous group [11,12]. Therefore, there is no consensus on the indications and the optimal subjects for surgical resection. This study retrospectively investigated whether the number of involved lymph nodes is associated with prognosis in patients that underwent surgery for p-stage III-N2 NSCLC. Identifying patients who receive survival benefit from surgical resection will positively contribute to determining the optimal treatment strategies.

### Patients and Methods

The hospital records of 690 consecutive patients who underwent a complete resection of NSCLC between 1995 and 2005 were reviewed. There were 469 patients with N0 disease, 84 with N1 disease, and 137 patients with N2 disease. This study focused on 137 patients with p-stage III/N2 NSCLC. Ten patients that underwent preoperative chemotherapy or radiation, and 6 patients underwent segmentectomy or partial resection of the lung were excluded. The preoperative assessments included chest roentgenography and computed tomography (CT) of the chest, and upper abdomen. The clinical N2 status was defined by the presence of a lymph node more than 1 cm in the short axis diameter. Bone scintigraphy was performed to detect bone metastasis. MRI (magnetic resonance imaging) of the brain was routinely employed for assessment of distant metastasis. Bronchoscopy was routinely performed to obtain a pathological diagnosis by transbronchial lung biopsy, and to evaluate endobronchial staging. The patients' records, including their clinical data, preoperative examination results, details of any surgeries, histopathological findings, and the TNM stages of all patients were also reviewed.

The patients underwent lobectomy, bilobectomy or pneumonectomy were enrolled in this study. A complete mediastinal lymphadenectomy was routinely performed. After surgery, en bloc dissected tissues were separated into each lymph node precisely. All resected specimens, including the primary tumor and resected hilar and mediastinal lymph nodes, were examined to determine both the tumor histology and the extent of lymph node metastases. Intraoperative frozen sections were examined if invasion of the tumor was suspected at the surgical margins. The histopathological findings were classified according to the World Health Organization criteria, and the UICC TNM staging system (7th edition) was also employed [5,13]. We investigated the association between total number of involved lymph nodes including hilar and mediastinal lymph nodes, and survival. We also investigated the association between skip mediastinal lymph nodes metastasis and survival. Skip metastasis was defined as mediastinal lymph nodes metastasis without hilar lymph nodes metastasis.

Postoperative systemic chemotherapy was performed for patients with stage III disease if the patients could tolerate such treatment after surgery, or unless the patients refused additional chemotherapy. The chemotherapy regimen used was carboplatin + paclitaxel, or carboplatin + gemcitabine.

Follow-up information was obtained from all patients through office visits or telephone interviews either with the patient, with a relative, or with their primary physicians. The patients were evaluated every 3 months by chest roentgenography, and chest CT scans and bone scintigraphy were performed every 6 months for the first 2 years after surgery and annually thereafter. The mean duration of observation was 57 months.

The survival curve was calculated by the Kaplan-Meier method, and the data were compared using the Log-rank test for a univariate analysis. Prognostic factors were analyzed by a multivariate analysis using Cox's proportional hazard model to adjust for potential confounding factors. Categorical variables were compared by Fisher's exact test. The differences were considered to be significant, if the p value was less than 0.05. The StatView V software package (Abacus Concept, Berkeley, CA) was used for all statistical analyses.

## Results

There were 121 patients who underwent either a pneumonectomy, bilobectomy or lobectomy for p-stage III/N2 NSCLC. The patients included 89 males and 32 females (Table 1). The mean age of the patients was 67.2 years (range: 44-85). One hundred patients (82.6%) had a smoking habit. The histological types included 65 adenocarcinomas (53.7%), 39 squamous cell carcinomas (32.2%) and 13 large cell carcinoma (10.7%) and 4 adenosquamous carcinomas (3.3%). There were 27 patients in T1, 53 in T2, 32 in T3, and 9 in T4 (stage IIIB). Clinical stages were diagnosed as stage I in 46 patients, stage II in 6, and stage III in 69. A pneumonectomy was performed in 25 (20.7%), a bilobectomy in 9 (7.4%), and a lobectomy in 87 (71.9%). The average number of dissected lymph nodes (N1 and N2) was 23.8 (range: 6-55). The average number of involved lymph nodes (N1 and N2) was 5.9 (range: 1-23). Skip mediastinal lymph nodes metastasis (N1 negative) was demonstrated in 41 patients (33.8%), and mediastinal lymph nodes metastasis with N1 disease (N1 positive) was found in 80 patients. The number of metastatic lymph nodes in patients with skip mediastinal lymph nodes metastasis was 1 in 17 patients, 2 in 7 patients, and ≥ 3 in 17 patients.

**Table 1:** Characteristics of the patients at p-stage III/N2

Average of age (range)	67.2 (44-85)
Gender; male/female	89/32
Histology	
Adenocarcinoma	65
Squamous cell carcinoma	39
Large cell carcinoma	13
Adenosquamous cell carcinoma	4
T factor	
T1	27
T2	53
T3	32
T4	9
Clinical Stage	
I	46
II	6
III	69
Operative procedure	
Operative procedure	
Pneumonectomy	25
Bilobectomy	9
Lobectomy	87
Number of involved lymph nodes (N1 +N2)	
1	17
2	21
3	19
4	10
≥5	54

**Figure 1:**
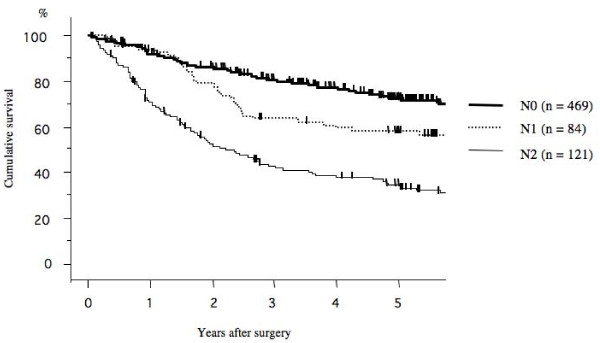
**Overall survival curves after surgery according to the pathological N factor**. The 5-year survival rate after surgery according to the pathological N stage (N0, N1, and N2) was 72.3%, 58.1%, and 33.4%, respectively.

**Figure 2:**
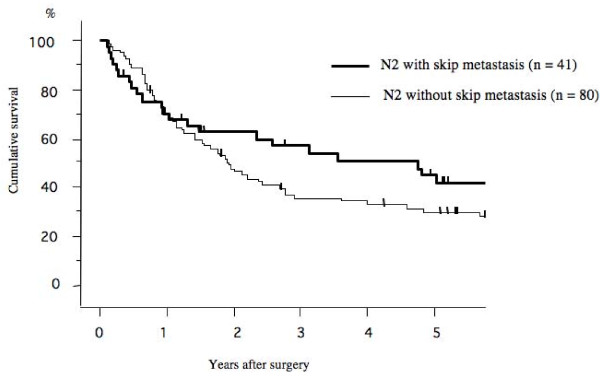
**Overall survival curves after surgery in p-N2 patients with/without skip metastasis**. The 5-year survival rate of skip mediastinal lymph nodes metastasis (41.7%) was better than that of mediastinal lymph nodes metastasis (N2) with N1 disease (30.1%). However, there was no significant difference (p = 0.216).

**Figure 3:**
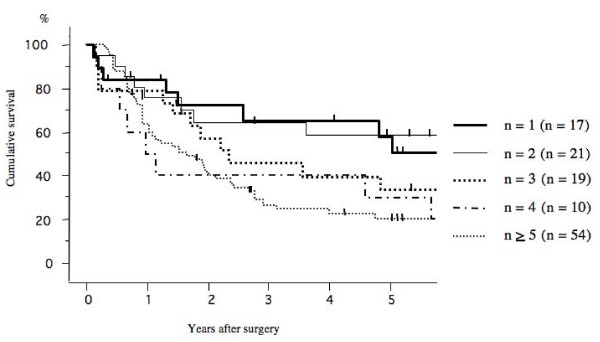
**Overall survival curves after surgery in p-N2 patients according to the number of involved lymph nodes**. The 5-year survival rate of the patients was 51.0% for single lymph node positive, 58.9% for 2 lymph nodes positive, 34.2% for 3 lymph nodes positive, 30.0% for 4 lymph nodes positive, and 20.4% for more than 5 lymph nodes positive.

**Figure 4:**
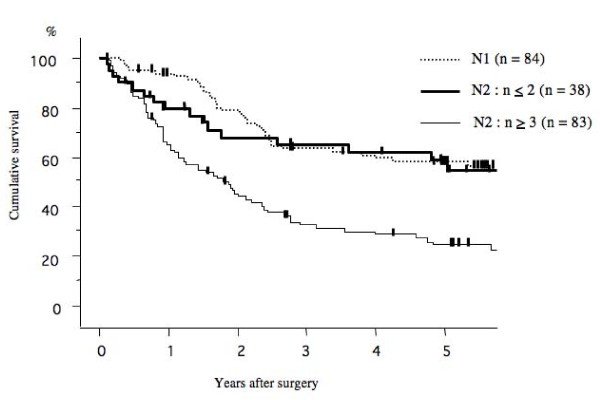
**Comparison of the overall survival between p-N1 patients and p-N2 patients with 2 or less than 2 involved lymph nodes**. There was no significant difference between the patients with N1 disease and the patients with 2 or less than 2 lymph nodes metastasis.

The 5-year survival rate after surgery according to the pathological N stage (N0. N1. and N2) was 72.3%, 58.1%, and 33.4%, respectively (Figure 1). Among, the 5-year survival rate of patients with mediastinal lymph node metastasis (N2) that had skip mediastinal lymph nodes metastasis (without N1 lymph node metastasis) was 41.7%; however that of mediastinal lymph nodes metastasis (N2) with N1 disease was 30.1% (Figure 2). The prognosis of patients with skip mediastinal lymph nodes metastasis showed a better tendency for survival, but there was no significant difference (p = 0. 216). As regards the total number of involved lymph node, the 5-year survival rate of the single lymph node positive patients was 51.0%, 58.9% for 2 lymph nodes positive, 34.2% for 3 lymph nodes positive, and 30.0% for 4 lymph nodes positive, and 20.4% for more than 5 lymph nodes positive (Figure 3). The patients with either single or 2 lymph nodes positive had a significantly better prognosis than the patients with more than 5 lymph nodes positive. The 5-year survival rate in the patients with 2 or less than 2 lymph nodes positive was 55.2%, whereas the 5-year survival rate in the patients with more than 2 lymph nodes positive was 24.7% (p < 0.001; Figure 4). There was no significant difference between the patients with N1 disease and the patients with 2 or less than 2 lymph nodes metastasis.

A univariate analysis of survival in patients with stage III NSCLC showed that T factor (T1 vs. T2-4, p = 0.0125), Surgical procedure (pneumonectomy or bilobectomy vs. lobectomy, p = 0.0345) and number of involved lymph nodes (≤2 vs. 3 ≤, p = 0.0041) were significant prognostic factors (Table 2). A multivariate analysis using these significant variables (T factor, surgical procedure, and number of involved lymph node) showed that the hazard ratio of the number of involved lymph node was 0.456 (95% confidence interval 0.265 - 0.785, p = 0.0046), thus indicating that it is a significant independent prognostic factor for p-stage III/N2 NSCLC (Table 3).

**Table 2:** Survival of patients with p-stage III/N2 NSCLC by a univariate analysis (log-rank test)

	n	Survival (%)	p value
Age			
<75 years	91	32.0	0.3519
≥ 75 years	30	39.5	
Gender			
Male	89	33.0	0.3981
Female	32	40.2	
T factor			
T1	27	47.8	0.0125
T2-4	94	30.8	
Histology			
Adenocarcinoma	63	41.0	0.0637
Others	58	28.2	
Surgical procedure			
Pneumonectomy/bilobectomy	34	20.7	0.0345
Lobectomy	87	40.1	
Skip metastasis			
Yes	41	41.7	0.2156
No	80	30.1	
Number of involved lymph nodes			
1 or 2	37	55.2	0.0041
3≤	84	24.7	

**Table 3:** Multivariate Cox proportional hazard analysis of the overall survival

Factors	relative risk	95% confidence interval	p value
T factor (T1 vs T2-4)	0.521	0.285 - 0.953	0.0344
Surgical procedure ((Pneumonectomy/bilobectomy vs Lobectomy)	0.676	0.417 - 1.097	0.1131
Number of involved lymph nodes (l or 2 vs 3≤)	0.456	0.265 - 0.785s	0.0046

## Discussion

NSCLC represents one of the most common and aggressive solid tumors, and it is difficult to cure. Reducing the mortality of lung cancer is an important public health issue. The status of lymph nodes is critical in planning treatment strategies if there is no distant metastasis. A complete surgical resection is considered to be the first line treatment for individuals with stage I-II NSCLC. However, more than half of the patients with NSCLC are diagnosed with N2-3 or M1 disease [14]. While chemotherapy for patients with advanced NSCLC prolongs survival and improves their quality of life, the majority of advanced stage patients succumb to disease within 2 years, thus, there is room for improvement [15]. The Japanese Lung Cancer Registry Study of 6644 resected NSCLC cases in Japan reported the 5-year survival rate for patients with stage IIIA and IIIB were 32.8% and 30.4%, respectively [10]. The 5-year survival rate for the patients with p-stage III was 33.4% in the current series, which was consistent with the Japanese Lung Cancer Registry Study. The indications for surgical treatment has remained mostly unchanged for a few decades. However, optimal therapeutic selection for stage III NSCLC is controversial. Presentations of stage III NSCLC range from apparently resectable tumors with single nodal metastasis to unresectable, bulky multi-station nodal disease, necessitating different treatment strategies. These heterogeneous subsets of stage III patients have been observed in a wide variety of clinical trials incorporating various combinations of chemotherapy, radiotherapy, and surgery. The evidence of whether surgical treatment for stage III/N2 disease improves the prognosis is unclear.

Skip metastasis is defined as the presence of mediastinal lymph node metastasis (N2 disease) without intra-lobar or hilar nodal involvement (N1). The mechanism of skip metastasis is thought to be direct subpleural lymphatic spread to the mediastinum. The incidence of skip N2 metastases is 20% to 40% of all N2 diseases, but the nature and clinical significance remain unclear [16]. Some investigators report that skip metastatic disease is a favorable N2 subset, possibly because it is usually associated with single-level N2 metastatic involvement [17,18]. The phenomenon of skip metastasis was pathologically identified in 41 of the current patients (33.8%). The patients with skip metastasis showed a better tendency for survival, but there was no significant survival difference between patients with skip metastasis compared to those without (p = 0. 216).

The present study focused on the number of involved lymph nodes in regional lymph node (N1 and N2). The 5-years survival rates of the patients at stage III/N2 was decreased according to the total number of involved lymph node (N1 + N2). The patients with 2 or fewer lymph node metastases had significantly better prognosis than those with 3 or more lymph node metastasis. Previous investigators demonstrated the single N2 disease showed favorable prognosis that multiple N2 disease [12,19]. However, the present study indicated prognostic information concerning subpopulation of patients with 2 or fewer lymph node metastases. The prognosis of patients with N2 lymph node metastasis in 2 or fewer nodes was comparable to the results of patients with N1 disease. In the multivariate analysis, T factor and the number of involved lymph node are also significant independent prognostic factors for patients with p-stage III/N2 NSCLC.

Several investigators demonstrated the effectiveness of induction chemotherapy [20]. Most studies report that surgical resection is recommended only for patients with mediastinal downstaging after chemotherapy, and not all patients with persistent mediastinal disease will benefit from surgery [21,22]. Clinical restaging is often inaccurate and appropriate selection of patients to undergo surgical resection following induction therapy is critical [23]. Lobectomy may be safely performed following induction therapy while pneumonectomy may carry a high and possibly unacceptable rate of perioperative mortality [23,24]. Decaluwé et al. suggested that the baseline single level N2 disease is an independent prognostic factor for long-term survival in surgical multimodality treatment [25]. Stupp et al. reported that neoadjuvant chemotherapy and radiotherapy followed by surgery in selected patients with stage IIIB NSCLC was feasible, and their 5-years survival was 40%, indicating it was comparable to the results of combined treatment for stage IIIA disease [26].

The present status of postoperative adjuvant chemotherapy for completely resected stage IIIA NSCLC is recommended based on the results of the large-scale phase III trials, using cisplatin-based regimens, such as IALT and ANITA studies, and a recent individual patient meta-analysis [27-29]. The clinical value of postoperative radiotherapy (PORT) in stage N2 non-small-cell lung cancer (NSCLC) is controversy [30]. Postoperative radiotherapy may be considered for fit patients with completely resected NSCLC with N2 nodal involvement, preferably after the completion of adjuvant chemotherapy [31]. A large multi-institutional randomized trial of PORT in these patient populations is now underway.

This retrospective study tried to clarify the prognostic importance of the number of involved lymph nodes in patients with p-stage III/N2 NSCLC who underwent complete dissection of the mediastinal lymph nodes. In conclusion, patients with 2 or fewer nodal involvement have the greater chance for cure and surgery has a significant role in their treatment. Patients with multi-station disease are frequently not amenable to complete resection and may be best approached with definitive chemotherapy and radiation. However, it is not possible to estimate exact number of lymph nodes by using current staging technique including mediastinoscopy and endobronchial ultrasound guided transbronchial fine needle aspiration cytology. The optimal incorporation of surgery into the multimodal approach therefore requires further clinical investigations in patients with p-stage III/N2 NSCLC.

## Conflict of interest statement

The authors declare that they have no competing interests.

## Authors' contributions

TH conceived of the study, and drafted the manuscript. MT participated in the study and performed the statistical analysis. SO, YS, YN, HS, HU, and FT participated in the study and coordination. All authors read and approved the final manuscript.
